# Differences in functional network between focal onset nonconvulsive status epilepticus and toxic metabolic encephalopathy: application to machine learning models for differential diagnosis

**DOI:** 10.1007/s11571-022-09877-0

**Published:** 2022-09-03

**Authors:** Seong Hwan Kim, Hayom Kim, Jung Bin Kim

**Affiliations:** Department of Neurology, Korea University Anam Hospital, Korea University College of Medicine, Seoul, Republic of Korea

**Keywords:** Network, Nonconvulsive status epilepticus, Toxic metabolic encephalopathy, Machine learning, Differential diagnosis

## Abstract

We aimed to compare network properties between focal-onset nonconvulsive status epilepticus (NCSE) and toxic/metabolic encephalopathy (TME) during periods of periodic discharge using graph theoretical analysis, and to evaluate the applicability of graph measures as markers for the differential diagnosis between focal-onset NCSE and TME, using machine learning algorithms. Electroencephalography (EEG) data from 50 focal-onset NCSE and 44 TMEs were analyzed. Epochs with nonictal periodic discharges were selected, and the coherence in each frequency band was analyzed. Graph theoretical analysis was performed to compare brain network properties between the groups. Eight different traditional machine learning methods were implemented to evaluate the utility of graph theoretical measures as input features to discriminate between the two conditions. The average degree (in delta, alpha, beta, and gamma bands), strength (in delta band), global efficiency (in delta and alpha bands), local efficiency (in delta band), clustering coefficient (in delta band), and transitivity (in delta band) were higher in TME than in NCSE. TME showed lower modularity (in delta band) and assortativity (in alpha, beta, and gamma bands) than NCSE. Machine learning algorithms based on EEG global graph measures classified NCSE and TME with high accuracy, and gradient boosting was the most accurate classification model with an area under the receiver operating characteristics curve of 0.904. Our findings on differences in network properties may provide novel insights that graph measures reflecting the network properties could be quantitative markers for the differential diagnosis between focal-onset NCSE and TME.

## Introduction

The differential diagnosis between nonconvulsive status epilepticus (NCSE) and toxic/metabolic encephalopathy (TME) has been a challenging clinical issue (Bearden et al. 2008; Kaplan and Birbeck 2006) because the two conditions share a common presentation, including loss of consciousness and unresponsiveness. The mainstay of NCSE treatment is the use of antiepileptic drugs (AEDs), whereas that of TME management is the correction of medical derangement or cessation of causative toxic drugs without necessarily using AEDs. In addition to the difference in treatment strategies for both conditions, timely differential diagnosis between NCSE and TME is critical for promoting a favorable prognosis through proper therapeutic intervention, considering the adverse effects of unnecessary use of AEDs in TME.

Although differential diagnosis based on clinical features is not difficult if there is evidence of the use of toxic agents or severe metabolic derangements (e.g., liver or kidney failure, lithium intoxication) (Angel and Young 2011), it is entirely dependent on electroencephalography (EEG) findings if there is no evidence of definite metabolic disturbance or potential toxic drug intake. Triphasic waves (TWs), now referred to as generalized periodic discharges (GPDs) with triphasic morphology (Hirsch et al. 2013, 2021) have been regarded as typical EEG findings of TME (Fernandez-Torre and Kaplan 2021). GPD patterns are also associated with active or terminal phases of NCSE; therefore, several morphologic characteristics of GPD that are associated with an ictal pattern have been proposed to distinguish it from TW, which is associated with a nonictal pattern (Bicchi et al. 2021). However, TWs had only a fair interrater agreement (kappa = 0.33) among 11 experts in a recent study, and moderate interrater agreement (kappa = 0.58) among 49 raters in another study, suggesting that reliance on differential diagnosis based on the morphologic patterns of EEG may be limited (Foreman et al. 2016; Gaspard et al. 2014). Considering the limitation of distinguishing NCSE from TME using the EEG morphology, development of differential diagnostic EEG markers for classification, rather than morphological characteristics, is required.

Although the pathophysiology of TME varies according to the underlying etiology, it has been widely accepted that all forms of TME have a common mechanism of altered function of the ascending reticular activating system and its projections to the cortex, leading to impairment of arousal and/or awareness (Posner et al. 2007). Therefore, cortical dysfunction and the generation of GPDs in the TME might occur in widespread areas simultaneously, rather than in focal regions. However, the postictal or interictal EEG patterns of focal-onset NCSE can appear as periodic discharges with a single or multifocal focus in terms of pathophysiological mechanisms, although EEG findings are often difficult to distinguish from GPDs in the TME on visual interpretation of EEG. Given the presence of focality in focal-onset NCSE, we hypothesized that hyperexcitable and relatively stable regions might be delineated during interictal or postictal periods of focal-onset NCSE, and that the functional network property in focal-onset NCSE might be more segregated than that of TME (van Diessen et al. 2013). Brain network analysis using EEG is widely used as a tool to objectively and quantitatively identify neurological abnormalities that could not be measured by conventional methods (Mehdizadehfar et al. 2020; Yi et al. 2022; Kim et al. 2022). Herein, we aimed to compare EEG network properties between focal-onset NCSE and TME during periods of periodic discharges using graph theoretical analysis. In addition, if there were differences in network properties, we sought to evaluate the applicability of graph theoretical measurements representing characteristic network properties as markers for differential diagnosis between focal-onset NCSE and TME using machine learning algorithms.

## Methods

### Subjects

This study was based on a retrospective review of an inpatient long-term video-EEG monitoring database between January 2020 and December 2020. From the entire database, we selected EEG data from patients who were confirmed to have focal-onset NCSE and TME through comprehensive evaluation, including electroclinical diagnosis by board-certified epileptologists (HK, JBK), neuroimaging studies, and laboratory examinations. The inclusion criteria for focal-onset NCSE were as follows: (1) proven ictogenic focus, (2) EEG findings according to the Salzburg criteria for NCSE (Leitinger et al. 2015, 2016), and (3) recovery of consciousness after treatment with AEDs. The inclusion criteria for TME were as follows: (1) decreased consciousness associated with exposure to toxic substances and/or metabolic abnormalities documented by laboratory examinations, such as elevated liver enzymes, elevated creatinine and blood urea nitrogen levels, electrolyte imbalance, glucose abnormality, and septicemia; (2) recovery of consciousness level after cessation of causative toxic substances and/or improvement of medical derangement without use of AEDs; (3) EEG findings that do not meet the Salzburg criteria for NCSE; and (4) patients without underlying epilepsy. The study followed the ethical guidelines of the Declaration of Helsinki and was approved by the institutional review board of the Korea University Anam Hospital (No. 2020AN0435).

## EEG recording and selection of periodic discharges

**Fig. 1:**
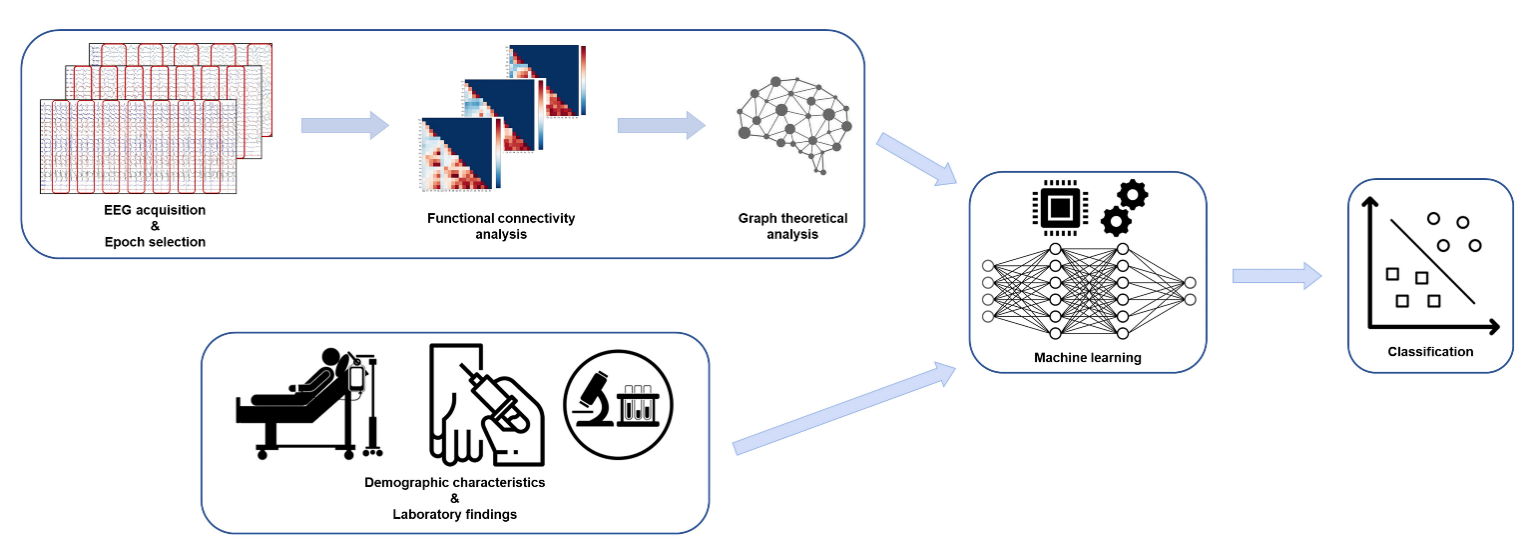
Flow diagram for the development of the differential diagnosis model between focal onset nonconvulsive status epilepticus and toxic/metabolic encephalopathy

An overview of this study is shown in Fig. 1. EEG recordings were conducted using a 32-channel recording system (Comet-PLUS, Grass Technologies Inc., West Warwick, RI, USA) with electrodes placed according to the International 10–20 system. EEG data were sampled at 200 Hz, and the bandpass filter was set between 0.1 and 70 Hz. Two board-certified epilepsy specialists (HK and JBK) visually inspected whole EEG recordings and carefully selected 10 non-consecutive 2 s epochs composed of periodic discharges for each patient.

The purpose of this study was to differentiate between focal-onset NCSE and TME through network analysis, using only EEG epochs showing periodic discharges that were difficult to distinguish visually. For the purpose of this study, we extracted EEG data from only epochs in which continuous nonictal periodic discharges that did not show changes in amplitude and frequency lasted more than 10 min, and excluded epochs that showed the following patterns suggesting ictal findings that can be distinguished even by visual inspection: (1) waxing and waning amplitude and frequency of EEG rhythm, (2) continuous ictal activity punctuated by low-voltage flat periods, and (3) evolving patterns leading to electrographic ictal activity (Treiman, Walton, & Kendrick, 1990).

## Network analysis

Detailed methods of functional connectivity and graph theoretical analyses have been described in our previous studies (Choi and Kim 2021; Kim et al. 2021). Functional connectivity was evaluated using coherence. Coherence analysis and visualization were performed using tailored Python scripts and the MNE-Python package (version 0.24.0) (Gramfort et al. 2013). Epochs were then bandpass-filtered into the following frequency bands: delta (0.1–4 Hz), theta (4–8 Hz), alpha (8–13 Hz), beta (13–30 Hz), and gamma (30–50 Hz). Subsequent analyses were performed separately for each band. Graph theoretical analyses were carried out on the EEG functional connectivity networks of patients with focal-onset NCSE and those with TME using the Brain Connectivity Toolbox (http://www.brain-connectivity-toolbox.net) and the BRAPH toolbox (http://braph.org) (Mijalkov et al. 2017; Rubinov and Sporns 2010).

A graph is an abstract representation of a network, consisting of a set of nodes and a set of edges. The presence of an edge between two nodes indicates the presence of interactions or connections between the nodes. The adjacency matrix contains information regarding the connectivity properties of the graph. Descriptions for global measures of graph theoretical analysis are presented in Table 1 (Rubinov and Sporns 2010). Network properties were characterized using a weighted undirected network model of graph theoretical analysis to avoid the arbitrariness of threshold selection for producing an adjacency matrix and to preserve the continuous nature of the correlated information (Rubinov and Sporns 2010). All graph measures were compared between focal-onset NCSE and TME using nonparametric tests with 1000 permutations. Statistical significance was set at *p* < .05, and corrected for multiple comparisons using the false discovery rate (FDR).

**Table 1:** Descriptions for global measures of graph theoretical analysis

Graph theoretical measures	Description
average degree	the average number of connections linked directly to a node
strength	average of sum of the weights of all edges connected to each node
radius	minimum eccentricity of all nodes
diameter	maximum eccentricity of all nodes
eccentricity	average of maximal distance between a certain node and any other node
characteristic path length	the extent of overall routing efficiency of a network
global efficiency	degree of efficiency at which information is propagated through the entire network
local efficiency	degree of efficiency at which information is propagated over a node’s direct neighbors
clustering coefficient	the extent of local clustering or cliquishness of a network
transitivity	ratio of total number of triangles to the number of triplets in the graph
modularity	the extent to which the nodes can be divided into several subsets with dense connections within but few connections between them
assortativity	correlation coefficient between the degrees/strengths of all nodes on two opposite ends of an edge
small-worldness	the extent of a graph having a similar characteristic path length as a random graph with the same degree distribution but significantly more clustered

## Machine learning applications

Demographic variables and laboratory findings, as well as global measures of graph theoretical analysis, were found to be possible markers for differentiating focal-onset NCSE and TME in group comparisons selected for use as input features in machine learning algorithms. To evaluate the utility of the selected input features to discriminate between the two conditions, eight different traditional machine learning methods were implemented using Python’s Orange toolbox (v.3.30.1) (Demšar et al. 2013): gradient boosting, logistic regression, random forest, support vector machine (SVM), naïve Bayes, neural network, k-nearest neighbors, and decision tree. For hyperparameter optimization, random search method with 3-fold cross validation was performed using 75% of the data as training set. The performance of each classifier was evaluated using a confusion matrix containing the parameters of positive predictive value, negative predictive value, sensitivity, specificity, accuracy, and F1 score, as follows:

Positive predictive value = TP / (TP + FP).

Negative predictive value = TN / (TN + FN).

Sensitivity = TP / (TP + FN).

Specificity = TN / (TN + FP).

Accuracy = (TP + TN) / (TP + TN + FP + FN).

F1 score = (2 x positive predictive value x sensitivity) / (positive predictive value + sensitivity).

where TP, FP, TN, and FN refer to the number of true positives, false positives, true negatives, and false negatives, respectively. Sensitivity and specificity were computed to generate receiver operating characteristic (ROC) curves. The area under the curve (AUC) for ROC was also computed. The Shapley Additive Explanations (SHAP) method was applied to the classifier model, showing the most accurate performance in evaluating and ranking the contribution of each variable to the model (Lundberg et al. 2020).

## Results

### Demographics and clinical characteristics

Clinical characteristics, including demographics, underlying chronic diseases, vital signs on EEG acquisition, and laboratory findings, are detailed in Table 2. Patients with TME were more likely to be older and female, as well as have chronic liver disease, than patients with NCSE (all *p* < .05). Patients with TME had lower platelet counts, lower level of serum magnesium, higher levels of blood urea nitrogen, creatinine, and aspartate aminotransferase than patients with NCSE (all *p* < .05).

**Table 2:** Demographic characteristics and laboratory findings

	NCSE (n = 50)	TME (n = 44)	*p*-value
Age (years)	66.12 ± 17.43	73.52 ± 13.13	0.021
Sex (women, %)	22 (44.0%)	32 (72.7%)	0.005
Hypertension (n, %)	23 (46.0%)	25 (56.8%)	0.295
Diabetes (n, %)	21 (42.0%)	18 (40.9%)	0.915
Chronic kidney disease (n, %)	5 (10.0%)	11 (25.0%)	0.053
Chronic liver disease (n, %)	2 (4.0%)	9 (20.4%)	0.013
Alcohol (bottle/week)	0.26 ± 1.14	0.32 ± 1.23	0.813
Smoking (pack years)	4.70 ± 12.63	4.29 ± 10.37	0.867
Systolic blood pressure (mmHg)	130.92 ± 20.70	135.95 ± 31.15	0.366
Diastolic blood pressure (mmHg)	76.36 ± 14.79	80.41 ± 25.45	0.341
Heart rate (/min)	90.14 ± 20.71	89.02 ± 21.49	0.797
Body temperature (°C)	36.90 ± 0.60	38.39 ± 9.72	0.279
Respiratory rate (/min)	18.82 ± 3.90	19.64 ± 4.64	0.357
Hemoglobin (g/dL)	10.96 ± 2.64	10.13 ± 2.70	0.135
White blood cell (x10^3^/µL)	11.27 ± 4.40	9.49 ± 5.31	0.078
Platelet (x10^3^/µL)	214.88 ± 104.49	144.14 ± 105.13	0.002
C-reactive protein (mg/L)	51.76 ± 66.24	69.84 ± 80.89	0.248
Sodium (mEq/L)	137.38 ± 6.94	139.57 ± 8.09	0.161
Potassium (mEq/L)	3.87 ± 0.52	4.18 ± 1.14	0.093
Calcium (mg/dL)	8.60 ± 0.94	8.82 ± 1.75	0.476
Magnesium (mg/dL)	1.09 ± 0.54	0.85 ± 0.16	0.010
Phosphorus (mg/dL)	3.50 ± 1.37	3.58 ± 1.68	0.825
Blood urea nitrogen (mg/dL)	21.67 ± 14.13	49.80 ± 36.30	< 0.001
Creatinine (mg/dL)	1.09 ± 1.00	2.23 ± 3.20	0.028
Aspartate transaminase (IU/L)	38.24 ± 51.56	123.18 ± 230.53	0.021
Alanine transaminase (IU/L)	33.32 ± 40.41	49.00 ± 87.51	0.258
Gamma-glutamyl transpeptidase (IU/L)	75.61 ± 124.64	86.14 ± 110.82	0.729
Total bilirubin (µmol/L)	1.12 ± 1.61	2.03 ± 2.59	0.050
Glucose (mg/dL)	164.43 ± 63.78	237.32 ± 294.63	0.115
Creatine phosphokinase (CPK, IU/L)	619.26 ± 2901.25	473.23 ± 1962.63	0.807

*NCSE* nonconvulsive status epilepticus, *TME* toxic/metabolic encephalopathy

Values are presented as mean ± standard deviation. Group comparisons were performed using independent *t*-test or chi-square test, where appropriate

## Graph theoretical analysis

**Fig. 2:**
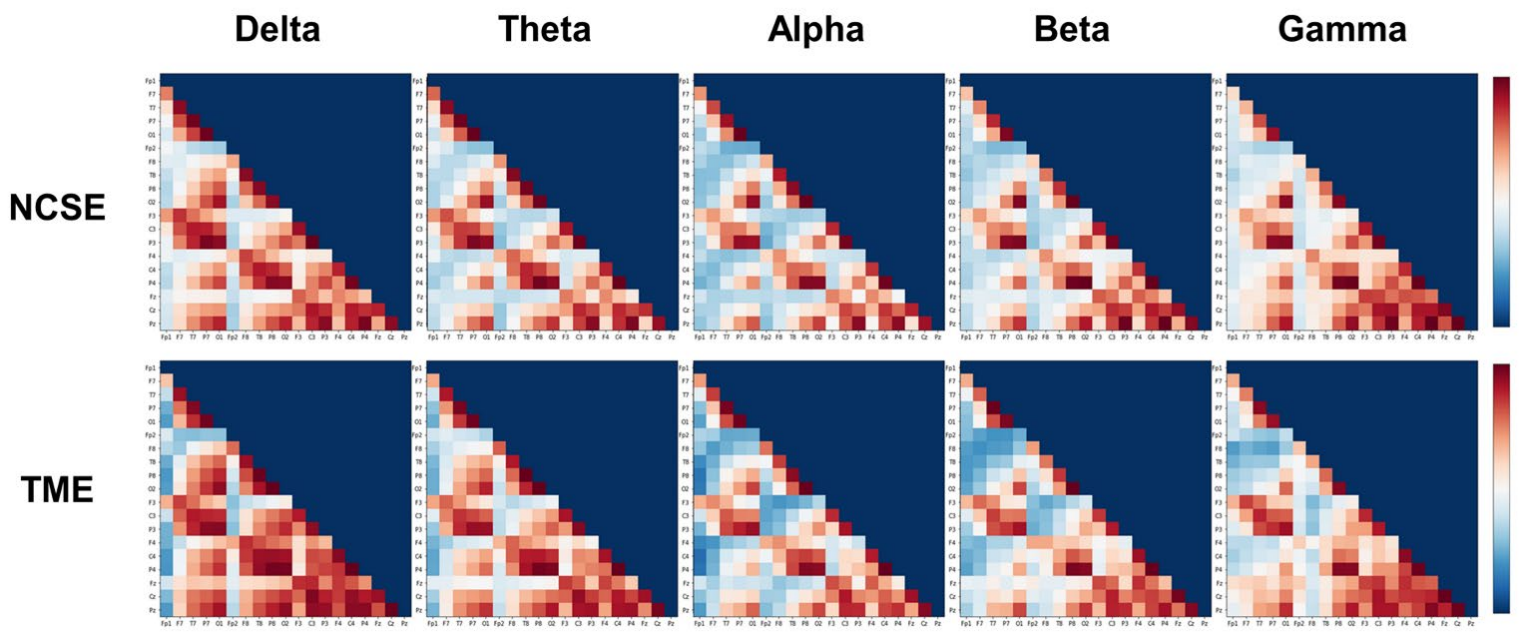
Functional connectivity in terms of coherence. The plots show adjacent matrices of the coherence between 19 pairs of scalp electroencephalography electrodes in each frequency band in focal onset nonconvulsive status epilepticus (NCSE, upper) and toxic/metabolic encephalopathy (TME, low)

Functional connectivity, in terms of coherence, is represented with adjacent matrices in Fig. 2. Comparisons of global graph measures between TME and NCSE are presented in Table 3. Average degree (in delta, alpha, beta, and gamma bands), strength (in delta band), global efficiency (in delta and alpha bands), local efficiency (in delta band), clustering coefficient (in delta band), and transitivity (in delta band) were higher in patients with TME than in those with NCSE (FDR-corrected *p* < .05). Patients with TME showed lower modularity (in delta band) and assortativity (in alpha, beta, and gamma bands) than those with NCSE (FDR-corrected *p* < .05).

**Table 3:** Results of graph theoretical analysis

Graph measurements	Delta		Theta		Alpha		Beta		Gamma	
	NCSE	TME	NCSE	TME	NCSE	TME	NCSE	TME	NCSE	TME
Degree	**11.436**	12.388*	10.570	11.103	**9.909**	10.844*	**10.613**	11.653*	**11.591**	12.919*
Strength	**5.598**	6.616*	5.236	5.701	4.494	5.153	4.823	5.448	6.023	6.598
Radius	7.551	5.485	5.928	5.430	16.164	6.159	17.223	34.099	63.784	25.249
Diameter	11.696	9.620	9.888	9.091	21.470	10.471	24.406	42.192	73.294	32.022
Eccentricity	8.087	7.234	7.787	7.095	18.322	8.022	18.099	37.106	34.161	24.809
Characteristic path length	3.512	3.189	3.769	3.441	5.231	3.741	5.814	7.051	7.815	5.040
Global efficiency	**0.406**	0.457*	0.395	0.422	**0.354**	0.390*	0.354	0.385	0.399	0.424
Local efficiency	**0.913**	1.108*	0.886	0.968	0.724	0.832	0.765	0.843	1.006	1.022
Clustering coefficient	**0.361**	0.406*	0.350	0.368	0.304	0.332	0.314	0.339	0.377	0.393
Transitivity	**0.573**	0.651*	0.546	0.575	0.482	0.527	0.516	0.551	0.631	0.641
Modularity	**0.219**	0.169*	0.263	0.241	0.271	0.232	0.222	0.190	0.163	0.132
Assortativity	0.199	0.143	0.222	0.165	**0.253**	0.194*	**0.213**	0.146*	**0.148**	0.058*
Small-worldness	0.837	0.858	0.841	0.848	0.797	0.851	0.747	0.810	0.724	0.646

Bold font with asterisk (^*^) represents statistically significant differences between focal-onset NCSE and TME. *NCSE* nonconvulsive status epilepticus, *TME* toxic/metabolic encephalopathy

## Machine learning performance & identification of feature importance

**Fig. 3:**
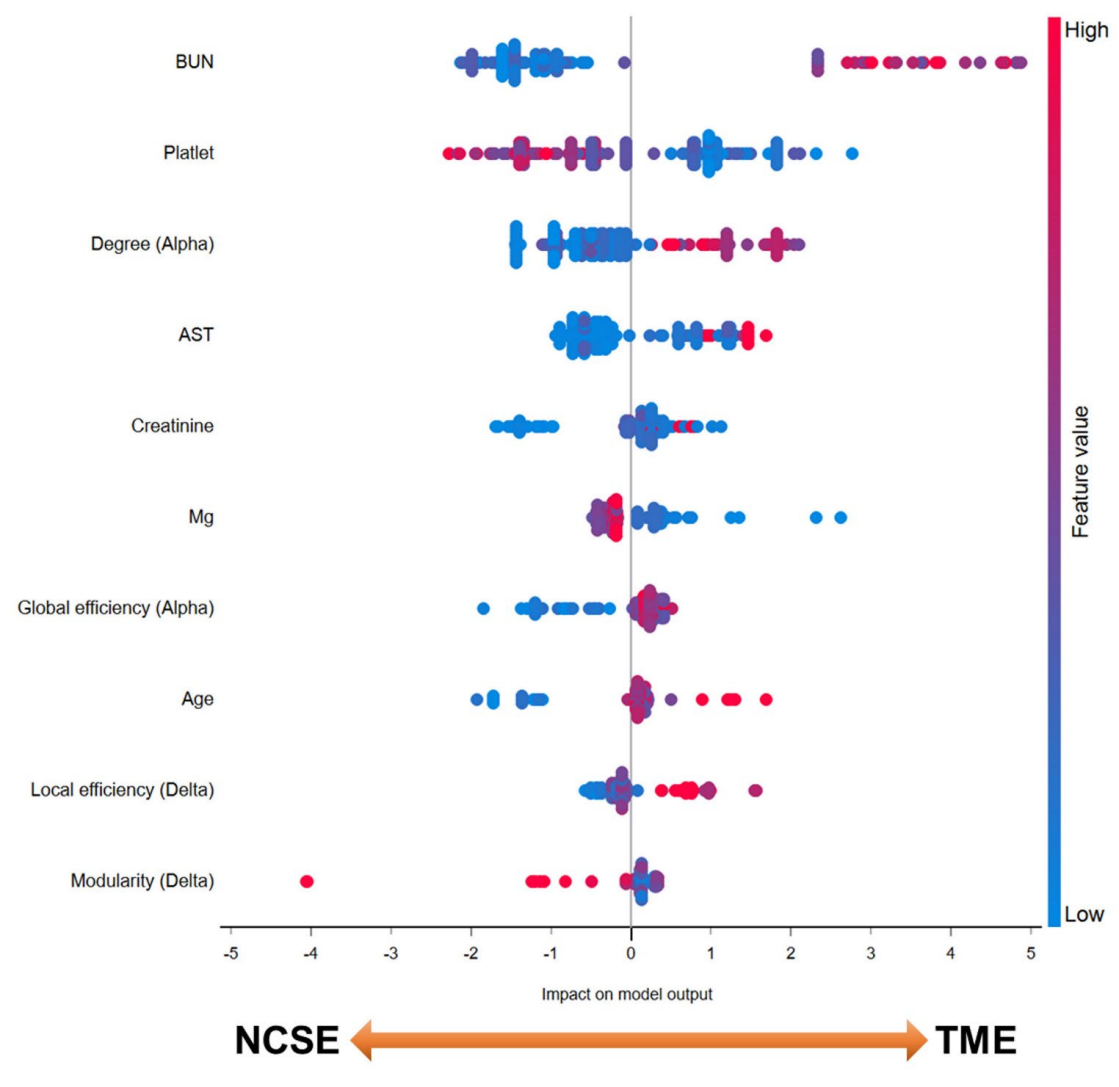
Receiver operating characteristics (ROC) curves of machine learning models. Each value presents the average result of 3-fold cross validation *FP* false positive, *kNN* k-Nearest Neighbors, *SVM* support vector machine, *TP* true positive

A total of 22 variables were selected for use as input features in machine learning algorithms, and they consisted of eight clinical variables (i.e., age, sex, presence of chronic liver disease, platelet counts, levels of serum magnesium, blood urea nitrogen, creatinine, and aspartate aminotransferase) and 14 graph measures, which were found to be possible features to discriminate the two conditions in the group comparisons. The performance and the ROC curves of each machine-learning algorithm are presented in Table 4; Fig. 3, respectively. Among the eight algorithms for classification, gradient boosting was the most accurate, with an AUC value of 0.904, classification accuracy of 0.833, F1 score of 0.834, positive predictive value of 0.835, negative predictive value of 0.812, sensitivity of 0.833, and specificity of 0.865.

**Table 4:** Performance of machine learning models. Each value presents the average result of 3-fold cross validation

Model	AUC	Accuracy	F1 score	PPV	NPV	sensitivity	specificity
Gradient Boosting	0.904	0.833	0.834	0.835	0.812	0.833	0.865
Logistic Regression	0.874	0.736	0.736	0.744	0.715	0.736	0.794
Random Forest	0.864	0.764	0.764	0.772	0.767	0.764	0.795
Support Vector Machine	0.820	0.722	0.718	0.725	0.733	0.722	0.711
Naïve Bayes	0.818	0.708	0.709	0.716	0.691	0.708	0.765
Neural Network	0.812	0.722	0.718	0.725	0.733	0.722	0.711
k-Nearest Neighbors	0.804	0.736	0.717	0.740	0.718	0.736	0.778
Decision Tree	0.698	0.681	0.681	0.684	0.667	0.681	0.722

*AUC* area under the receiver operating characteristics curve, *PPV* positive predictive value, *NPV* negative predictive value

**Fig. 4:**
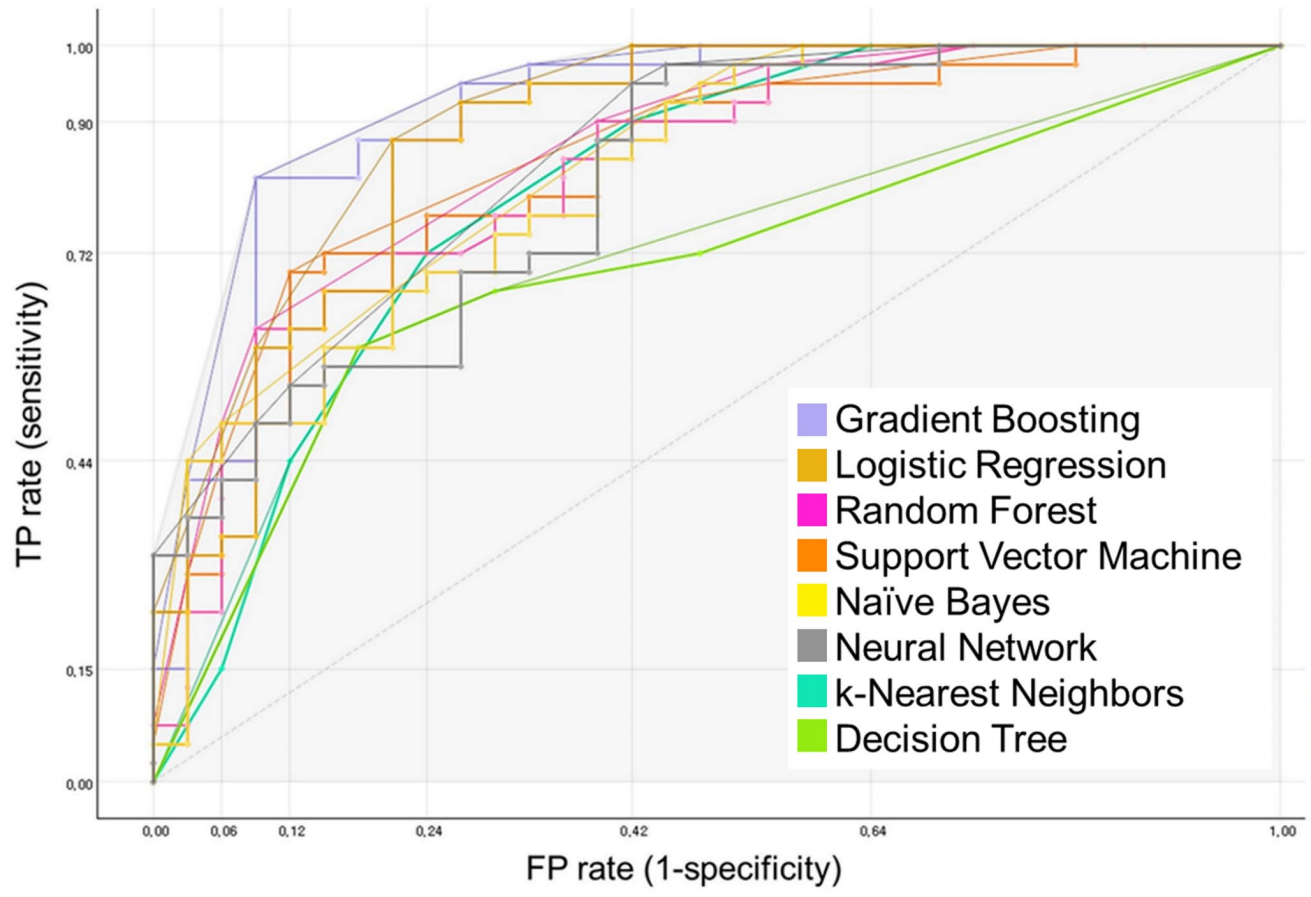
Shapley Additive Explanations (SHAP) model The top 10 most contributing features to the gradient boosting model are presented *AST* Aspartate transaminase, *BUN* blood urea nitrogen, *Mg* magnesium, *NCSE* nonconvulsive status epilepticus, *TME* toxic/metabolic encephalopathy

Figure 4 shows the SHAP importance matrix plots, which depict the power and direction of the top 10 most contributing features to the gradient boosting model. A high blood urea nitrogen level had the greatest impact on the model output to predict TME. Following the high blood urea nitrogen level, in the order of low platelet count, high average value in the alpha band, high aspartate aminotransferase level, high creatinine level, low magnesium level, high global efficiency value in the alpha band, old age, high local efficiency value in the delta band, and low modularity value in the delta band, were found to be important contributing features in the gradient boosting model to predict TME.

## Discussion

We investigated the differences in EEG network properties during periods of periodic discharges between focal-onset NCSE and TME. The major findings were as follows: (1) the property of EEG functional connectivity was more integrated and efficient in TME, relative to NCSE; (2) machine learning algorithms, including the EEG global graph measures as input features, could classify the NCSE and TME with high accuracy, and gradient boosting was the most accurate classification model with an AUC > 0.9; and (3) in addition to laboratory findings suggesting metabolic derangement, EEG graph measures reflecting integrated and efficient network properties (i.e., high degree and global efficiency in the alpha band, high local efficiency, and low modularity in delta band) were found to be contributing features in the gradient boosting model to predict TME.

Recent advances in graph theoretical network analysis enable the assessment of the topological architecture of complex human brain networks (Farahani et al. 2019; Sporns 2013b). Therefore, graph theoretical analysis has been widely applied to quantify characteristics of functional network in terms of efficiency and integration in various neurological disorders, as well as conditions affecting brain function (Bullmore and Sporns 2009; Choi and Kim 2021; Kim et al. 2021; Park et al. 2020; van Diessen et al. 2013). Our findings of relatively high degree, strength, and global efficiency in TME suggest that network properties of TME might be more integrated and efficient, relative to those of focal-onset NCSE (Bullmore and Sporns 2009). Modularity is regarded as a global graph measure of network segregation (Sporns 2013a), which is calculated by partitioning a network into groups of modules with high connectivity within modules relative to the connectivity between regions in distinct modules (Girvan and Newman 2002; Newman 2006). In addition, high assortativity could be interpreted as high-degree nodes have a high tendency to connect with each other (Bullmore and Sporns 2009). Taken together, the higher modularity and assortativity found in focal-onset NCSE compared with that in TME suggest that network properties of focal-onset NCSE may tend to be segregated into functionally stable regions and irritative zones having strong local connection around the ictogenic focus (Bialonski and Lehnertz 2013), whereas those of TMEs may be globally integrated (Sporns 2013a).

The mechanism underlying the more integrated and efficient network properties in patients with TME, relative to those with NCSE, is not fully understood. It has been widely accepted that GPDs with triphasic morphology, the EEG hallmark of TME, may originate from dysfunction of the thalamocortical circuits with recruitment of metabolically impaired cortical neurons (Karnaze and Bickford 1984). Based on the hypothesis regarding the generation of periodic discharges in the TME, it is plausible that the simultaneous involvement of pathophysiological mechanisms in widespread brain areas might explain the altered EEG network with highly synchronized and efficient properties in the TME. There are dynamic changes in network topology in patients with seizures (van Diessen et al. 2013). The ictal period could be characterized by a more synchronized and integrated network configuration of the epileptic brain (Ponten et al. 2009; Schindler et al. 2008; van Diessen et al. 2013). The number of connections gradually declines to preictal levels after the midictal phase, resulting in a less synchronizable and disintegrated network topology after ictal termination (Ponten et al. 2009; Schindler et al. 2008; van Diessen et al. 2013). Therefore, our findings of a more segregated network property may be in accordance with the network configuration of postictal or interictal periods of focal-onset NCSE. Given that focal-onset NCSE and TME may have different pathophysiologies in terms of network, graph theoretical measures could be considered as important biomarkers to differentiate focal-onset NCSE and TME.

We found that machine learning algorithms based on EEG graph measures could classify focal-onset NCSE and TME with relatively high accuracy. These findings suggest that quantitative analysis indicators that utilize network properties can complement the limitations of the visual interpretation of EEG for differential diagnosis between the two conditions. Indeed, differences in graph measures between focal-onset NCSE and TME were observed predominantly in the delta band, suggesting the presence of invisible pathophysiological differences within periodic discharges with delta frequency between the conditions. Furthermore, the accuracy of differential diagnosis was beyond 90% using only the short EEG data for 20 s, thus implying that the graph theoretical analysis could be an optimal framework for quantitatively differentiating network properties between focal-onset NCSE and TME, and that graph measure-based machine learning algorithms may be clinically useful for distinguishing the conditions.

Among the variables, four EEG graph measures (i.e., degree in the alpha band, global efficiency in the alpha band, local efficiency in the delta band, and modularity in the delta band) played important roles in the differential diagnosis, which were included in the top 10 most contributing features. Considering that laboratory findings may differ according to the causative condition of TME, EEG graph measures could contribute to the machine learning model as pivotal features to consistently differentiate between focal-onset NCSE and TME. To verify the accuracy of differential diagnosis of the machine learning algorithm using EEG graph measures and to determine the contributing power of EEG graph measures, a subgroup analysis of the TME for each causative condition is required with a larger study population.

The present study had several limitations that should be considered when interpreting the results of this study. First, the sample size was relatively small. Second, we could not externally validate the accuracy of machine learning models. Therefore, the generalizability of the accuracy of the differential diagnosis between focal-onset NCSE and TME might be limited, although internal validation has been statistically performed. Third, as an inherent limitation of the retrospective study design, we could not identify the difference in network properties according to the change in severity in patients with TME. Further longitudinal studies are required to develop a machine learning algorithm for the differential diagnosis between focal-onset NCSE and TME by confirming the changes in network properties associated with the severity of TME. Finally, there are various etiologies of the development of NCSE (Lee et al. 2021); therefore, the property of the NCSE network might overlap with that of TME when the occurrence of NCSE is due to metabolic derangement. Additional analyses are required to determine whether there are distinct network characteristics that can be used for the differential diagnosis of each cause of NCSE.

One strength of our study is that EEG global graph measures were not dependent on the location of the ictogenic focus. Therefore, the proposed gradient boosting model based on EEG global graph measures in our study could be applied in general to distinguish focal-onset NCSE from TME, regardless of the location of ictogenic focus. The application of an interpretable and explainable model is another strength of our study. The model enables physicians to recognize what the important clinical and EEG variables are, which can promote the clinical usefulness of applying the model as a decision support system in the differential diagnosis between focal-onset NCSE and TME. It may also provide insights for further studies to understand the mechanisms of generating periodic discharges in the TME and focal-onset NCSE from the perspective of functional networks.

## Conclusion

Our results suggest that network properties may differ between TME and focal-onset NCSE. Patients with TME had a more integrated and efficient network, whereas those with focal-onset NCSE had segregated network properties. Using graph measures reflecting the differences between the conditions, machine learning models could classify TME and focal-onset NCSE with an accuracy higher than 90%. Our finding on differences in network properties may provide novel insights into the distinct pathophysiological mechanisms underlying the generation of periodic discharges in focal-onset NCSE and TME. Furthermore, EEG global graph measures could be considered as quantitative markers for differential diagnosis between focal-onset NCSE and TME.

## Data Availability

Data are available from the corresponding author upon reasonable request.
